# Changes in Bone Marrow Fat upon Dietary-Induced Weight Loss

**DOI:** 10.3390/nu12051509

**Published:** 2020-05-22

**Authors:** Manuela Spurny, Yixin Jiang, Solomon A. Sowah, Ruth Schübel, Tobias Nonnenmacher, Robert Bertheau, Romy Kirsten, Theron Johnson, Jens Hillengass, Christopher L. Schlett, Oyunbileg von Stackelberg, Cornelia M. Ulrich, Rudolf Kaaks, Hans-Ulrich Kauczor, Tilman Kühn, Johanna Nattenmüller

**Affiliations:** 1 Heidelberg University Hospital, Diagnostic and Interventional Radiology, Im Neuenheimer Feld 110, 69120 Heidelberg, Germany; Manuela.Spurny@med.uni-heidelberg.de (M.S.); Yixin.Jiang@med.uni-heidelberg.de (Y.J.); ruth.schuebel@gmx.de (R.S.); Tobias.Nonnenmacher@med.uni-heidelberg.de (T.N.); Robert.Bertheau@med.uni-heidelberg.de (R.B.); oyunbileg.stackelberg@med.uni-heidelberg.de (O.v.S.); Hans-Ulrich.Kauczor@med.uni-heidelberg.de (H.-U.K.); 2 German Cancer Research Center (DKFZ), Division of Cancer Epidemiology, Im Neuenheimer Feld 581, 69120 Heidelberg, Germany; s.sowah@dkfz-heidelberg.de (S.A.S.); romy.kirsten@nct-heidelberg.de (R.K.); t.johnson@Dkfz-Heidelberg.de (T.J.); r.kaaks@Dkfz-Heidelberg.de (R.K.); t.kuehn@dkfz-heidelberg.de (T.K.); 3 Roswell Park Comprehensive Cancer Center, Elm & Carlton Streets, Buffalo, New York 14263, USA; Jens.Hillengass@RoswellPark.org; 4 Department of Diagnostic and Interventional Radiology, Medical Center-University of Freiburg, Faculty of Medicine, University of Freiburg, Hugstetter Straße 55, D-79106 Freiburg, Germany; christopher.schlett@uniklinik-freiburg.de; 5 Huntsman Cancer Institute and Department of Population Health Sciences, University of Utah, 2000 Circle of Hope, Salt Lake City, UT 84112-5550, USA; neli@hci.utah.edu

**Keywords:** magnetic resonance imaging, bone marrow fat content, diet induced weight loss, obesity, overweight

## Abstract

Background: Bone marrow fat is implicated in metabolism, bone health and haematological diseases. Thus, this study aims to analyse the impact of moderate weight loss on bone marrow fat content (BMFC) in obese, healthy individuals. Methods: Data of the HELENA-Trial (Healthy nutrition and energy restriction as cancer prevention strategies: a randomized controlled intervention trial), a randomized controlled trial (RCT) among 137 non-smoking, overweight or obese participants, were analysed to quantify the Magnetic Resonance Imaging (MRI)-derived BMFC at baseline, after a 12-week dietary intervention phase, and after a 50-week follow-up. The study cohort was classified into quartiles based on changes in body weight between baseline and week 12. Changes in BMFC in respect of weight loss were analysed by linear mixed models. Spearman’s coefficients were used to assess correlations between anthropometric parameters, blood biochemical markers, blood cells and BMFC. Results: Relative changes in BMFC from baseline to week 12 were 0.0 ± 0.2%, −3.2 ± 0.1%, −6.1 ± 0.2% and −11.5 ± 0.6% for Q1 to Q4. Across all four quartiles and for the two-group comparison, Q1 versus Q4, there was a significant difference (*p* < 0.05) for changes in BMFC. BMFC was not associated with blood cell counts and showed only weaker correlations (<0.3) with metabolic biomarkers. Conclusion: Weight loss is associated with a decrease of BMFC. However, BMFC showed no stronger associations with inflammatory and metabolic biomarkers.

## 1. Introduction

Overweight and obesity are major health problems of societies in industrial countries, but also in emerging nations. From 1975 to 2014, obesity increased by the factor 6 worldwide, to 640 million adults [1]. Consequences of overweight and obesity, which are defined as body mass index (BMI) equal to or more than 25 kg/m^2^ and 30 kg/m^2^ respectively, are cardiovascular and musculoskeletal diseases, diabetes mellitus type 2, different types of cancer [1,2], and benign and malign haematological diseases [3].

Bone marrow fat (BMF) consists of adipocytes and forms the bone marrow, together with haematopoietic and stromal cells [4,5]. Haematopoietic stem cells stand at the beginning of blood cell production, while mesenchymal stem cells produce adipocytes and bone cells. They are embedded in reticulum cells as surrounding structure. The older human individuals get, the more adipocytes replace the blood cell production within the bone marrow, leading to a physiological increase in bone marrow fat content (BMFC) parallel to increasing age. The distribution of BMFC in the spine also depends on physical activity [6]. The more individuals are active, the less BMFC is found in the spine [6]. After subcutaneous adipose tissue (SAT) and visceral adipose tissue (VAT), BMF is the third largest fat depot of the body [5], which also accounts for the largest cell population within the bone marrow. However, bone marrow adipocytes are regarded as different entities, apart from white and brown adipocytes [7,8], implying that BMF cannot be easily compared to other fat depots. Recent studies showed that its function goes beyond the mere filling of inert space [9]. BMF, rather, plays a complex role in energy storage, bone metabolism, the endocrinological system and is even involved in the regulation of bone metastases [5,10]. Furthermore, BMF seems to play a crucial role in the development of haematologic diseases like multiple myeloma, aplastic anaemia [11], and leukaemia [5] and is suspected to partly promote their progression [12]. As BMF is linked to metabolism and haematologic diseases, it is important to obtain more understanding of this relationship by analysing the effects of changes in BMF following weight loss. Currently, only a few studies exist on weight loss and changes in BMF after bariatric surgery [13,14,15,16,17,18], and only rare studies exist about BMF after dietary-induced weight loss, not covering non-diabetics or both sexes [19,20]. Both studies about dietary-induced weight loss had only small numbers of participants, with 20 and 29 participants respectively, and shorter dietary programs over six and four weeks, respectively [19,20]. Thus, the HELENA-Trial (**He**a**l**thy nutrition and **en**ergy restriction as c**a**ncer prevention strategies: a randomized controlled intervention trial) is the first study that analyses BMFC after diet-induced weight loss after 12 weeks of intervention, and that also covers both sexes with a large number of participants (*n* = 150).

Therefore, the aim of this study was to investigate whether BMFC changes after moderate weight loss in a study population of obese or overweight, but otherwise metabolically healthy individuals. Furthermore, we assessed if there are associations between BMFC and VAT, liver fat content (LFC), blood cell counts, as well as between BMFC and biomarkers of lipid and glucose metabolism as well as inflammation.

To this end, we used data acquired in a nutrition intervention trial with an intervention phase of 12 weeks, followed by a maintenance and a follow-up phase of 38 weeks altogether. At baseline, after 12 weeks and after 50 weeks, Magnetic Resonance Imaging (MRI)-examinations were performed to measure the bone marrow fat content.

## 2. Materials and Method

### 2.1. Study Population

For this study, the data from participants of the HELENA-Trial (trial registration number: NCT02449148 ClinicalTrials.gov) [21], a randomised dietary intervention study at the German Cancer Research Centre (DKFZ), Heidelberg, which had been undertaken to study the effects of continuous versus intermittent calorie restriction between May 2015 and May 2017, were taken in a post hoc analysis to find out whether moderate weight loss influences BMFC. This study has been approved by the ethics committee of the medical faculty of the University of Heidelberg, Germany.

In brief, the study population consisted of 150 overweight or obese, non-smoking individuals (50% female) between 35 and 65 years without severe, chronic diseases (neither kidney nor liver dysfunction, no major cardiovascular disease) nor cancer in the past or present [21]. Other exclusion criteria were diabetes mellitus, HbA1c levels ≥ 6.5% and/or fasting glucose levels ≥ 126 mg/dL at screening [21]. There were no exclusion criteria for blood cell counts. At baseline, participants were randomly allocated to three groups, two intervention groups with either intermittent (ICR) or continuous calorie restriction (CCR), or to a control group (CG) [21]. The study phases included a 12-week intervention phase, a 12-week maintenance phase and a 26-week follow-up phase [21]. The aim of both intervention groups was to reduce net calorie intake by 20 percent per week [21]. The intermittent calorie restriction group should limit their net calorie intake to 25 percent of their required calorie intake on two not directly consecutive days per week and consume a healthy, eu-caloric diet on the other five days [21]. The continuous calorie restriction group should limit their calorie intake to 80 percent of the required calorie intake on seven days per week [21]. Thus, on average, both intervention groups had the same average calorie intake of 80% of their required calorie intake per week. Participants of the control group just were advised on a healthy diet according to the German Nutrition Society without any calorie restriction [21]. Written informed consent was obtained from each participant prior to enrolment [21].

MRI examinations were performed at baseline, after 12 weeks and after 50 weeks at the Department of Diagnostic and Interventional Radiology of the University Hospital Heidelberg. Exclusion criteria were the typical contraindications for MRI, e.g., claustrophobia, cardiac pacemakers or defibrillators, medical or metallic foreign bodies, that could not be removed and were not approved for 1.5 Tesla-MR, non-removable electronic implants or devices, joint prostheses, etc. [21].

MRI analyses for the present study were performed for 137 participants.

At baseline, after the intervention phase, after the maintenance phase and after the follow-up phase, the participants were characterized in detail by questionnaires and medical examinations, including blood draws [21].

The study design, methods and results of the main-study of the HELENA-Trial have been described in detail in previous publications [21,22].

### 2.2. Laboratory Methods

Blood-based biomarkers (glucose, HDL; high density lipoprotein, LDL; low density lipoprotein, cholesterol, triglycerides, HbA1c) and blood cell counts (erythrocytes, leukocytes, neutrophil granulocytes, lymphocytes, monocytes, thrombocytes) were measured in the central laboratory of Heidelberg University Hospital. Serum biomarkers (ALT; alanine aminotransferase, AST; aspartate aminotransferase, GGT; gamma-glutamyltransferase, insulin, CRP; C-reactive protein, IFN-γ, interferon gamma, TNF-α, tumor necrosis factor-alpha, IL-6; interleukin 6, IL-8; interleukin 8, LDH; lactate dehydrogenase, adiponectin, leptin) were measured with electrochemiluminiscence with a Quickplex SQ 120 (Meso Scale Discoveries, MD, USA) in the Division of Cancer Epidemiology at the German Cancer Research Centre (DKFZ), Heidelberg.

The laboratory analyses are described in detail in a previous publication [22].

### 2.3. Imaging

BMFC, LFC, VAT and subcutaneous adipose tissue were measured by a 1.5 Tesla MR scanner (MAGNETOM Aera, Siemens Healthcare, Erlangen, Germany). Hardware, MR-protocol and software remained constant for all MRI examinations.

**Figure 1:**
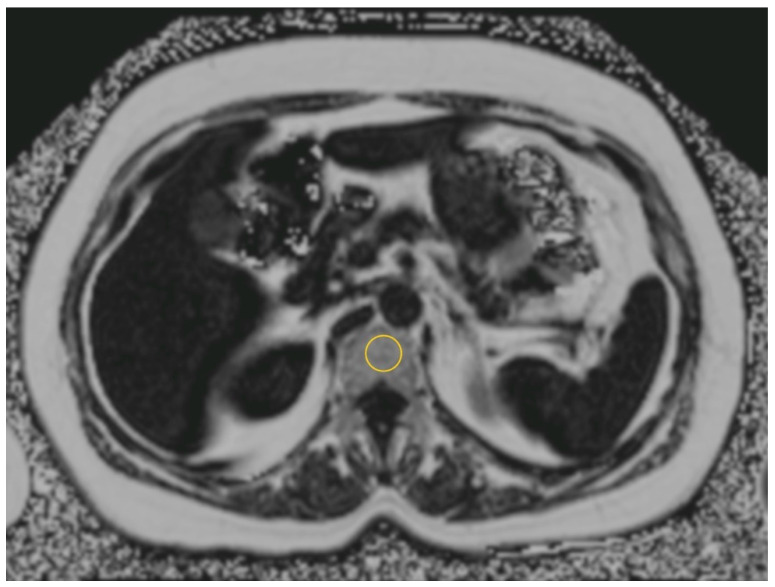
Assessment of bone marrow fat content in first lumbar vertebral body in the anterior part, medium level, avoiding vessels and the anterior border on a proton density fat fraction (PDFF) map with one region of interest with 2.0 cm^2^ (ROI, yellow circle).

The complete MRI examination for each time point took about 15 to 20 min. For the present analysis of the BMFC, axial images of the upper abdomen (1 block, epigastric region inclusive liver and lumbar vertebral body L 1 and 2) were acquired by using a multiecho Dixon sequence with 6 echoes and with T2* correction (FAT_QUANT_fl3d-vibe-Dixon; Siemens LiverLab, Siemens Healthcare, Erlangen, Germany; see Figure 1) with the following parameters: relaxation time 11.50 msec, echo time 2.39 msec, slice thickness of 4 mm, flip angle of 6.0°, signal-to-noise ratio 1.00 and voxel size in-plane 1.6 × 1.6 mm, with a field of view of 420 mm and an acquisition time of 24 s with breath-hold [21,23,24,25].

Regions of interest (ROI, 2 cm^2^) were manually placed in lumbar vertebral body 1 and 2 (anterior part in the medium level) on the proton density fat fraction (PDFF) map [26] by using a post-processing software (OsiriX, Pixmeo SARL, Bernex, Switzerland; see Figure 1). The mean value of lumbar vertebral body 1 and 2 was taken. Image analysis was obtained in a blinded manner with independent readers not aware of the study time point or group of each participant. For the placement of the ROI, vessels, lesions, the borders of the vertebral body and surrounding tissue (e.g., muscles, visceral adipose tissue) were avoided. The measurement was taken at the time points of baseline, week 12 and week 50 for each participant in a blinded manner.

Intra- and inter-reader coefficients of the measurements were assessed by Spearman’s rank correlation coefficient in 40 examinations and were 0.937 and 0.715, respectively (readers: M.S. and Y.J.).

The measurements of the liver fat content also using the PDFF map are described in detail elsewhere [27,28].

VAT and SAT were semi-automatically quantified by an in-house software (Medical Imaging Interaction Toolkit (MITK)) [21] using a 2-point dixon sequence covering the body from the neck to the upper part of the legs, as described in detail elsewhere [21,29].

### 2.4. Statistical Analyse

The primary study endpoint of the HELENA-Trial was to analyse whether intermittent calorie restriction is superior to continuous calorie restriction in respect to body composition, metabolism and adipose tissue gene expression. Recently published results by Schübel et al. have shown no significant differences between both intervention groups [22] (see Supplementary Table S1). For this post hoc analysis, the original study cohort was re-classified into weight loss quartiles irrespective of the intervention groups (ICR, CCR) or control group (see Supplementary Figure S1). Quartiles were created based on the weight loss after the 12-week intervention phase: Quartile 1 (≤2% weight loss, *n* = 35), quartile 2 (>2% and ≤4.5% weight loss, *n* = 34), quartile 3 (>4.5% and ≤7.5% weight loss, *n* = 35) and quartile 4 (>7.5% weight loss, *n* = 33). Linear mixed models with adjustment for sex and age were carried out to analyse the effects of weight loss on BMFC. Log-relative changes [30] were used to evaluate changes in body composition during the study.

Spearman’s coefficients were calculated to assess the associations between anthropometric parameters, blood biochemistry markers (ALT, AST, GGT, HDL, total cholesterol, triglycerides, fasting glucose, HbA1c), serum biochemistry markers (ALT, AST, GGT, insulin, CRP, IFN-γ, TNF-α, IL-6, IL-8, LDH, adiponectin, leptin), blood cells (erythrocytes, leukocytes, neutrophil granulocytes, lymphocytes, monocytes, thrombocytes) and BMFC, but also VAT, SAT and LFC. SAS Version 9.4 (Cary, NC, USA) was used for the statistical analyses.

## 3. Results

### 3.1. Characteristics of the Study Population at Baseline

The characteristics of the study population at baseline are given in Table 1. Some of the basic data are already published in a recent publication about the HELENA-Trial by the same group [31]. The BMFC at baseline was as follows: quartile 1: 43.9 ± 8.7%, quartile 2: 42.6 ± 8.3%, quartile 3: 44.6 ± 7.9%, quartile 4: 41.4 ± 10.2%. Height, weight, BMI, SAT and VAT were nearly similar across all four groups at baseline. The participants in Q4 were slightly younger (47.3 ± 8.5 years) than the participants from Q1 to Q3 (50.9 ± 6.4 years, 50.8 ± 8.4 years, 51.2 ± 7.9 years). The blood cell counts (erythrocytes, leukocytes, neutrophil granulocytes, lymphocytes, monocytes, thrombocytes) were within the physiological range and equally distributed in the four quartiles.

### 3.2. Correlations of Bone Marrow Fat in Comparison to Blood Biomarkers and Body Fat Volumes

Correlations of BMFC with weight, waist circumference, BMI, VAT, SAT and blood biomarkers, as well as blood cells (erythrocytes, leukocytes, neutrophil granulocytes, lymphocytes, monocytes, thrombocytes) at baseline, after 12 weeks and after 50 weeks are shown in Table 2. Neither for anthropometric measurements nor for blood biomarkers or blood cells counts like leukocytes or lymphocytes did we observe correlations at rho > 0.4 and *p* < 0.05 for BMFC.

A nominally statistically significant, but weaker correlation between BMFC and body weight, with rho = −0.21 (*p* = 0.0143 at baseline), was found. However, for week 12 and week 50, no such correlation was observed. Similarly, waist circumference at baseline was weakly associated with BMFC at baseline (rho = −0.25, *p* = 0.0041), but not at week 12. Also, BMI showed a weaker correlation at baseline with BMFC with rho = −0.31 and *p* < 0.01, which, however, was not apparent at week 12 and week 50.

Further cross-sectional correlations between BMFC and other covariates were weaker, with rho values < 0.3. Although some correlations were nominally statistically significant at single time points (i.e., adiponectin, HbA1c, VAT, SAT, Cholesterol, HDL, LDH, ALT, GGT), there was no consistent pattern across all time points (see Table 2).

### 3.3. Effects of Weight Loss on Bone Marrow Fat

**Figure 2:**
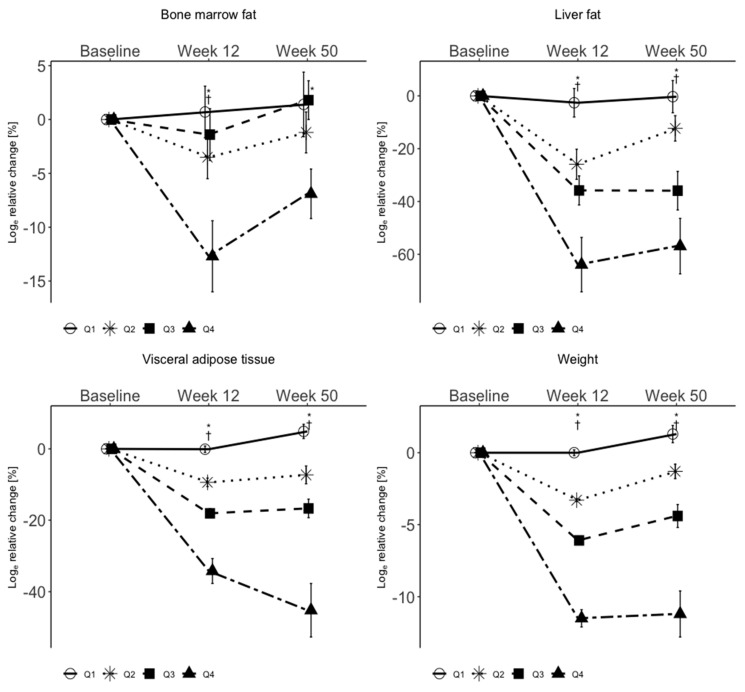
Relative changes of BMFC, LFC, VAT and weight by weight loss quartiles. BMFC, LFC, VAT and weight. Data are shown as mean ± SEM for Log relative change with baseline values as reference. Abbreviations: BMFC, bone marrow fat content; LFC, liver fat content; SEM, standard error of the mean; VAT, visceral adipose tissue; †, significant between the extreme quartiles 1 and 4; *, significant for all quartiles.

Relative changes in body weight and BMFC between baseline and week 12 and baseline and week 50 are shown in Table 3 and Figure 2. The data of weight in Table 3 and of weight, LFC and VAT in Figure 2 have already been published in a recent paper by the same group [31].

Relative changes in body weight between baseline and week 12 for Q1 to Q4 were as follows: 0.0% ± 0.2%, −3.2% ± 0.1%, −6.1% ± 0.2% and −11.5% ± 0.6%. From baseline to week 50, weight changes were, across the quartiles Q1 to Q4: 1.3% ± 0.6%, −1.3% ± 0.5%, −4.4% ± 0.8% and −11.2% ± 1.6%.

Relative changes in BMFC were, from baseline to week 12, 0.7% ± 2.4% among participants in Q1, −3.5% ± 2.0% in Q2, −1.4% ± 2.4% in Q3 and −12.7% ± 3.3% in Q4. At week 50, the relative changes in BMFC were 1.4% ± 3.0%, −1.2% ± 1.9%, 1.8% ± 1.8% and −6.9% ± 2.3% among participants in quartiles 1, 2, 3 and 4, respectively. Across all four weight loss quartiles and for the two-group comparison, Q1 versus Q4, there was a significant difference (*p* < 0.01) for changes in BMFC. For the two-group comparisons, Q1 versus Q2 and Q1 versus Q3, no significant differences (*p* > 0.05) were observed. The relative decrease in BMFC in Q4 up to the end of the intervention phase (−12.7% ± 3.3%) and to the follow-up phase (−6.9% ± 2.3%) was significantly greater compared to Q1, Q2 and Q3 (week 12: 0.7% ± 2.4%, −3.5% ± 2.0% and −1.4% ± 2.4%, week 50: 1.4% ± 3.0%, −1.2% ± 1.9% and −1.8% ± 1.8%). The relative change in BMFC across all quartiles was statistically significant not only for the end of the intervention phase, but also for the end of the follow-up phase (*p* < 0.01, baseline to week 12; *p* = 0.02 baseline to week 50) (see Table 3 and Figure 2).

## 4. Discussion

In this study, we analysed, in a post hoc manner, the effect of moderate dietary weight loss on BMFC in overweight and obese, but metabolically healthy individuals after an intervention phase of 12 weeks. We observed a decrease in BMFC with weight loss, with participants in the highest quartile of weight loss achieving a significantly greater reduction in BMFC (−12.7% after 12 weeks) in comparison to those in the lowest quartile (0.7% after 12 weeks). Therefore, we can conclude that it is possible to reduce BMFC with dietary-induced weight loss. However, a certain level of weight loss (>7.5% after 12 weeks for Q4) in overweight or obese individuals must be reached for a significant decrease of BMFC in our study population.

In the literature, a significant correlation was observed between significant weight loss and significant loss of BMFC in a study by Vogt et al., that examined a small group of 29 obese diabetics [19], which is in line with our results in 137 obese non-diabetics. Our findings are however in contrast to the study by Kim et al., which did not show significant BMFC loss six months after gastric bypass surgery of 30 obese women with or without diabetes, even if the patients had lost significant amounts of weight after the operation (27.3 ± 6.8 kg) [13], but Kim et al. detected a correlation between BMF and HbA1c in the diabetic women, who showed decreases in HbA1c parallel to decreases in BMFC. In our study, correlations between BMFC and HbA1c were very weak (rho < 0.2). In the study by Kim et al., BMFC was measured by magnetic resonance spectroscopy [13].

The study of Vogt et al. suggests that there are parallels between obese diabetics and non-diabetics [19], but those results are based on a significant weight loss (−13.2 kg after 15 weeks) from mean BMI of 34.0 kg/m^2^ to a BMI of 29.9 kg/m^2^ [19], which means a change from obese to overweight. In our study, the differences in BMI were less in comparison to Vogt et al. Only Q3 and Q4 showed changes in BMI from obese to overweight (Q3: 30.9 ± 3.4 at baseline, 29.1 ± 3.3 at T1, Q4: 31.6 ± 3.7 at baseline, 28.2 ± 3.3 at T1), whereas Q1 and Q2 remained obese at T1 (Q1: 32.1 ± 4.1 at baseline, 32.1 ± 4.2 at T1, Q2: 31.1 ± 3.7 at baseline, 30.1 ± 3.6 at T1).

A positive correlation between BMFC and LFC has been described for children with non-alcoholic fatty liver disease (NAFLD) [32] and for obese young adults between 19 and 45 years, in comparison to young adults of normal weight [33]. In obese adults, we could not detect such a correlation after moderate weight loss.

Further, we did not observe significant correlations between BMFC and insulin, although we found significant correlations between VAT and triglycerides, insulin and CRP, which has been published by our group recently [31]. LFC was also significantly correlated with insulin and ALT, which is also a predictive factor for NAFLD [27].

Bredella et al. described a positive association between serum lipid levels, especially triglycerides, and BMF in obese in comparison to normal weight individuals [33]. In our obese or overweight participants, we could detect a weak correlation between BMFC and cholesterol after 12 weeks and after 50 weeks. Furthermore, they found a negative correlation between BMFC and circulating HDL (r = −0.21, *p* = 0.019) [33]. While we also observed a statistically significant correlation between BMFC and HDL at baseline and after 50 weeks, this correlation was weak (rho = 0.21 at baseline and with r = 0.23 after 50 weeks) and not apparent at week 12 in the study.

We found weaker correlations between BMFC and adiponectin at baseline and after 12 weeks (rho = 0.20 and *p* = 0.0194 at baseline, rho = 0.21 and *p* = 0.0145 in week 12), which is also described in the research work of Cawthorn et al. [34], who found that a higher level of circulating adiponectin is correlated with an increased level of BMF during caloric restriction in a mouse model. However, the comparison between mice and humans is difficult, because BMFC and the distribution of BMF are different in mice from that in humans. Furthermore, Cawthorn at al. assessed BMF in their animal experiments from femora and tibiae, i.e., not from vertebral BMF [34]. Altogether, it is known that obesity is connected with circulating adiponectin [35,36,37]. So, our study shows, once more, that BMF is linked to metabolism, which may be underlined by the above-described weak correlation between BMFC and HbA1c and adiponectin, but we did not find correlations with inflammation markers, which are associated with adiponectin in the literature [36]. That our study also could not show any correlations between BMFC and glucose or insulin, but with adiponectin and HbA1c, is therefore surprising. One reason could be that adiponectin has complex functions besides the regulation of the effect of insulin, which is to be studied in experimental future studies. One other reason might be that we worked with metabolically healthy persons with a normal regulation of insulin metabolism. We also have to ask why we could find a weak correlation between BMFC and HbA1c. It is known that BMFC and HbA1c have a correlation in postmenopausal women with type 2 diabetes [38], even with a BMI < 25 kg/m^2^. Maybe, in metabolically healthy, but overweight individuals, HbA1c reacts earlier than insulin to changes of BMFC. It may also be dependent of the location of the BMF. Pham et al. [39] recently described that insulin has no effect on vertebral BMF, but on femoral BMF. So, maybe our results confirm the lacking effect on vertebral BMF.

We also could not detect a correlation between BMFC and blood cells (erythrocytes, leukocytes, neutrophil granulocytes, lymphocytes, monocytes, thrombocytes) in our study, which was not surprising, because we worked with healthy individuals, whose blood cells were within the physiological limits at baseline. To our knowledge, changes in blood cells after dietary interventions have not yet been compared to changes in BMFC in recent studies.

However, several previous studies have shown that there is an association between obesity and haematologic disorders like monoclonal gammopathy of undetermined significance (MGUS) and multiple myeloma (MM) [3,8]. The underlying pathophysiological mechanisms of this connection seem to be based on interactions of the clonal plasma cells with adipocytes and the cytokines they produce [8]. As a consequence of a high-fat diet, BMFC increases and decreases parallel to weight loss, as described in mouse models before [40], which is confirmed in our findings. Lwin et al. showed, in a mouse model, that a high-fat diet promotes MM, because the microenvironment in the bone is disturbed [41], whereas Ruan et al. showed that MM drives osteoblast progenitor cells to turn to adipogenesis [42]. Trotter et al. found that patients with MM have more preadipocytes and larger mature adipocytes [12], so that adiposity before MM might be protumourigenic [8]. Our study might support the hypothesis raised in these mouse models, that weight loss could help to restore the bone marrow microenvironment with a decrease of BMFC, which might help patients with MGUS to prevent the shift to MM by weight loss.

Another observation was made in respect of anorexia nervosa and fat distribution in the bone marrow. In the state of anorexia, i.e., extreme underweight, BMF increases [43,44,45,46], but decreases with recovery [47]. We found in our study that loss of bone marrow fat is dependent on a remarkable amount of weight loss, however the otherwise healthy individuals of our study are not readily comparable to anorectic individuals. On the other hand, Abella et al. described that there is a correlation between the amount of weight loss and increase of BMF in patients with anorexia nervosa [46], which was also confirmed by Cawthorn et al. [34]. Interestingly, in this study, the amount of circulating adiponectin increased with the amount of BMFC [34]. Therefore, it can be concluded that BMF has an equalizing function in respect of body weight. The increase of BMF in the study by Abella et al. was due to an increase of the diameter of adipocytes [46]. This finding suggests that in further studies with overweight or obese individuals, the diameter of adipocytes could be measured before and after weight loss to find out if weight loss triggers the reduction of the diameter of adipocytes. However, invasive bone marrow punctures would be necessary to investigate this, which we could not obtain in healthy individuals.

Another limitation of our study is that we did not perform H-magnetic resonance spectroscopy (H-MRS) to differentiate between saturated and unsaturated fat in the bone marrow. Patsch et al. found out that diabetes was associated with higher levels of saturated and lower levels of unsaturated BMF [48]. However, the multiecho dixon sequence with PDFF is a precise method to measure the fat content of organs or structures, which was first developed for measuring LFC [49], where it was found to be a robust method and an equivalent alternative to H-MRS [50]. One of the first studies to use PDFF for measuring BMFC was that of Gee et al., who examined the accuracy of PDFF measuring BMFC in the presence of trabecular bone and described the use of PDFF as an accurate method in the lumbar spine [51].

Finally, it should be noted that the present analyses were based on a post hoc classification of study participants by overall weight loss achieved in the study. However, there were no differential effects of the initial dietary interventions (CCR and ICR, Supplementary Figure S1) on BMFC (Supplementary Table S1), and thus, statistical adjustment for initial trial arms did not affect the present findings. For further details we refer to Schuebel et al. [22].

## 5. Conclusions

In conclusion, BMF decreases significantly in overweight and obese, but metabolically healthy individuals after dietary-induced weight reduction of more than 7.5%. In our study, BMFC showed no stronger associations with inflammatory and metabolic biomarkers nor blood cell counts. However, our results underline the role of BMF as a metabolically active fat depot. In addition, as obesity is a risk factor for some malignancies and BMFC is linked to bone health, metabolism and haematological diseases, the role and a potential preventive capacity of reduced BMFC after weight loss should be evaluated in future studies.

## Figures and Tables

**Table 1:** Characteristics of the four weight-loss groups (quartile (Q)1 to Q4) at baseline (T0), *n* = 137.

	Q1	Q2	Q3	Q4
	≤2%	>2% to ≤4.5%	>4.5% to ≤7.5%	>7.5%
	*n* = 35	*n* = 34	*n* = 35	*n* = 33
Women (*n* (%))	19 (54.3)	14 (41.2)	18 (51.4)	17 (51.5)
Age (year)	50.9 ± 6.4	50.8 ± 8.4	51.2 ± 7.9	47.3 ± 8.5
Height (cm)	171.4 ± 10.6	174.2 ± 9.8	173.8 ± 10.6	172.8 ± 7.9
Weight (kg)	94.8 ± 15.7	93.7 ± 14.4	93.9 ± 15.3	94.7 ± 14.4
Waist circumference (cm)	106.1 ± 12.0	104.9 ± 10.7	103.1 ± 10.8	103.5 ± 12.2
BMI (kg/m^2^)	32.2 ± 4.1	30.8 ± 3.6	31.0 ± 3.4	31.6 ± 3.6
VAT (L)	5.3 ± 2.2	4.8 ± 2.1	4.8 ± 2.0	4.7 ± 2.1
SAT (L)	13.1 ± 4.6	11.2 ± 2.9	12.1 ± 3.9	12.9 ± 4.1
BMFC (%)	43.9 ± 8.7	42.6 ± 8.3	44.6 ± 7.9	41.4 ± 10.2
LFC (%)	7.1 ± 4.4	8.8 ± 7.8	7.9 ± 6.5	7.4 ± 4.9
ALT (U/L)	25.0 ± 7.3	30.8 ± 13.9	26.6 ± 12.2	24.8 ± 9.9
AST (U/L)	21.7 ± 4.0	25.4 ± 6.8	22.3 ± 3.9	22.4 ± 5.2
GGT (U/L)	29.4 ± 14.0	25.5 ± 16.2	29.8 ± 19.7	24.3 ± 12.6
Triglycerides (mg/dL)	138.9 ± 65.7	135.0 ± 91.8	145.3 ± 94.2	109.4 ± 55.0
Cholesterol (mg/dL)	210.3 ± 33.9	201.9 ± 36.9	214.5 ± 36.5	203.3 ± 32.0
HDL (mg/dL)	53.5 ± 14.8	53.1 ± 14.5	56.3 ± 13.5	53.4 ± 15.3
LDL (mg/dL)	129.1 ± 26.2	120.2 ± 25.7	129.2 ± 26.8	128.0 ± 27.9
Glucose (mg/dL)	93.5 ± 8.0	93.2 ± 7.0	94.9 ± 6.9	91.9 ± 8.3
Insulin (mU/L)	14.9 ± 7.8	12.0 ± 6.9	10.9 ± 5.1	11.4 ± 5.6
HbA1c (%)	5.4 ± 0.4	5.5 ± 0.3	5.5 ± 0.3	5.5 ± 0.3
HOMA-IR	3.5 ± 1.9	2.8 ± 1.8	2.6 ± 1.2	2.6 ± 1.4
CRP (ng/pL)	7.0 ± 8.7	4.1 ± 5.5	3.8 ± 2.8	3.9 ± 3.8
IFN-γ (ng/µL)	16.6 ± 16.1	12.9 ± 12.9	17.5 ± 16.7	11.1 ± 7.8
TNF-α (ng/µL)	4.3 ± 2.7	4.0 ± 2.5	5.0 ± 2.6	4.2 ± 2.5
IL-6 (ng/µL)	2.0 ± 1.7	1.8 ± 3.5	1.3 ± 0.8	1.3 ± 1.1
IL-8 (ng/µL)	10.6 ± 4.4	14.2 ± 23.7	9.8 ± 4.8	10.6 ± 5.3
LDH (U/L)	197.3 ± 30.1	197.4 ± 26.8	192.7 ± 31.8	200.2 ± 28.3
Adiponectin (ng/mL)	15.6 ± 8.4	18.7 ± 11.4	16.9 ± 11.4	19.9 ± 13.7
Leptin (ng/mL)	29.2 ± 25.3	19.8 ± 20.3	21.5 ± 15.2	29.7 ± 29.4
Erythrocytes (1/pl)	4.8 ± 0.4	4.8 ± 0.5	4.9 ± 0.4	4.9 ± 0.3
Leukocytes (1/nl)	6.4 ± 1.5	6.1 ± 1.5	6.3 ± 1.3	6.3 ± 1.3
Neutrophil granulocytes (%)	58.9 ± 6.3	56.6 ± 8.1	57.7 ± 7.1	56.5 ± 9.2
Lymphocytes (%)	29.6 ± 6.2	30.6 ± 6.2	30.3 ± 7.1	31.5 ± 8.6
Monocytes (%)	6.1 ± 1.4	7.2 ± 2.0	6.6 ± 1.4	6.2 ± 1.5
Thrombocytes (1/nl)	239.2 ± 49.2	237.9 ± 50.6	240.5 ± 46.7	249.6 ± 60.5

Data are shown as mean ± standard deviation (SD). Abbreviations: ALT, alanine aminotransferase; AST, aspartat aminotransferase; BMFC, bone marrow fat content; BMI, body mass index; CRP, C-reactive protein; GGT, gamma glutamyl transferase; IFN-γ, interferon gamma; IL6, interleukin 6; IL8, interleukin 8; HbA1c, haemoglobin A1c; HDL, high-density lipoprotein; HOMA-IR, homeostatic model assessment for insulin resistance; LDH, lactatdehydrogenase; LDL, low-density lipoprotein; LFC, liver fat content; Q, quartile; SAT, subcutaneous adipose tissue; TNF-α, tumour necrosis factor-alpha; VAT, visceral adipose tissue.

**Table 2:** Spearman’s correlations between anthropometric parameters, metabolic parameters, inflammation parameters, blood cells and BMFC at baseline, after 12 weeks and after 50 weeks, *n* = 137.

	Bone Marrow Fat Content					
	Baseline		Week 12		Week 50	
	rho	*p*-Value	rho	*p*-Value	rho	*p*-Value
**Weight**	−0.21	0.0143*	0.02	0.7999	0.05	0.592
**Waist circumference**	−0.25	0.0041*	0.06	0.5434	-	-
**BMI**	−0.31	0.0003*	−0.12	0.177	0.01	0.8748
**VAT**	−0.007	0.9394	0.18	0.045*	0.19	0.032*
**SAT**	−0.20	0.0221*	−0.002	0.986	0.05	0.598
**LFC**	0.03	0.7228	0.17	0.0653	0.17	0.0699
**ALT**	0.10	0.237	0.25	0.0045*	0.22	0.0159
**AST**	0.03	0.7087	0.15	0.0917	0.11	0.255
**GGT**	0.12	0.1872	0.28	0.0018*	0.32	0.0004*
**Triglycerides**	0.02	0.8556	0.09	0.3463	0.14	0.1216
**Cholesterol**	0.13	0.1314	0.18	0.0471*	0.28	0.0024*
**HDL**	0.21	0.0166*	0.14	0.1165	0.23	0.0121*
**LDL**	0.10	0.2473	0.14	0.1203	0.14	0.1399
**Glucose**	0.03	0.699	0.005	0.9596	0.09	0.3087
**Insulin**	0.04	0.6538	0.11	0.2432	0.12	0.1863
**HbA1c**	0.007	0.9388	0.06	0.5014	0.19	0.044*
**HOMA-IR**	0.05	0.5945	0.10	0.2525	0.13	0.1641
**CRP**	−0.10	0.2636	0.12	0.1725	0.10	0.2824
**INF-g**	−0.03	0.7411	0.06	0.484	−0.01	0.913
**TNF-a**	−0.05	0.5489	0.01	0.8965	−0.09	0.2886
**IL-6**	−0.12	0.1729	−0.02	0.8467	0.03	0.7866
**IL-8**	0.17	0.0528	0.07	0.4171	0.07	0.4803
**LDH**	0.25	0.0048*	0.16	0.0746	-	-
**Adiponectin**	0.20	0.0194*	0.21	0.0145*	-	-
**Leptin**	−0.02	0.8133	0.14	0.1191	0.07	0.4251
**Erythrocytes**	−0.01	0.8797	0.03	0.7095	0.07	0.4519
**Leukocytes**	−0.09	0.3155	−0.009	0.9222	−0.05	0.569
**Neutrophil granulocytes**	0.00	0.9855	−0.08	0.3929	−0.06	0.4887
**Lymphocytes**	0.03	0.7398	0.07	0.4235	0.05	0.6114
**Monocytes**	−0.07	0.4249	0.03	0.7577	0.02	0.7993
**Thrombocytes**	−0.09	0.3041	−0.12	0.1895	0.05	0.5778

* Correlation is significant at the 0.05 level (two tailed). Abbreviations: ALT, alanine aminotransferase; AST, aspartat aminotransferase; BMFC, bone marrow fat content; BMI, body mass index; CRP, C-reactive protein; GGT, gamma glutamyl transferase; IFN-γ, interferon gamma; IL6, interleukin 6; IL8, interleukin 8; HbA1c, haemoglobin A1c; HDL, high-density lipoprotein; HOMA-IR, homeostatic model assessment for insulin resistance; LDH, lactatdehydrogenase; LDL, low-density lipoprotein, LFC, liver fat content; SAT, subcutaneous adipose tissue; TNF-α, tumour necrosis factor-alpha; VAT, visceral adipose tissue.

**Table 3:** Mean values and relative change (%) of weight and BMFC in week 12 and week 50 across the weight loss quartiles compared with the baseline values, *n* = 137.

		Baseline Mean ± SD	Week 12 Mean ± SD	Log_e_ Relative Change (Baseline–Week 12) Mean ± SEM	*p*-Value	Week 50 Mean ± SD	Log_e_ Relative Change (Baseline–Week 50) Mean ± SEM	*p*-Value
**Weight (kg)**	Q1	94.8 ± 15.7	94.8 ± 15.6	0.0 ± 0.2	<0.01*	96.1 ± 16.1	1.3 ± 0.6	<0.01*
	Q2	93.7 ± 14.4	90.7 ± 14.0	−3.2 ± 0.1		93.3 ± 13.9	−1.3 ± 0.5	
	Q3	93.9 ± 15.3	88.4 ± 14.4	−6.1 ± 0.2		89.8 ± 15.8	−4.4 ± 0.8	
	Q4	94.7 ± 14.4	84.4 ± 12.8	−11.5 ± 0.6		84.8 ± 14.2	−11.2 ± 1.6	
**BMFC (%)**	Q1	43.9 ± 8.7	44.0 ± 8.5	0.7 ± 2.4	<0.01*	44.3 ± 7.5	1.4 ± 3.0	0.02
	Q2	42.6 ± 8.3	41.3 ± 8.2	−3.5 ± 2.0		41.9 ± 7.5	−1.2 ± 1.9	
	Q3	44.6 ± 7.9	44.1 ± 8.3	−1.4 ± 2.4		46.3 ± 8.4	1.8 ± 1.8	
	Q4	41.4 ± 10.2	37.0 ± 10.8	−12.7 ± 3.3		38.6 ± 9.7	−6.9 ± 2.3	

* *p* = significant at the 0.05 level (two tailed). Data are shown as mean ± SD and as mean ± SEM for week 12 and week 50 for Log_e_ relative change with baseline values as reference. *p*-values of the four weight loss quartiles were calculated with linear mixed models adjusted for sex and age (baseline to week 12, baseline to week 50). Abbreviations: BMFC, bone marrow fat content; SD, standard deviation; SEM, standard error of the mean.

## Supplementary Materials

The following are available online at https://www.mdpi.com/2072-6643/12/5/1509/s1, Figure S1: Distribution of the three original study arms (ICR, CCR, controls) among the four weight loss quartiles after 12 weeks. CCR, continuous calorie restriction; Con, controls; ICR, intermittent calorie restriction, Table S1: Mean values and relative change (%) of BMFC week 12 and week 50 across the study arms ICR, CCR, and controls compared with the baseline values, n=137.
