# Supplementary material for: Changes in Bone Marrow Fat upon Dietary-Induced Weight Loss
Source: Nutrients. 2020 May 22;12(5):1509. doi: 10.3390/nu12051509 (PMC7284630; doi:10.3390/nu12051509)
Supplement: Supplementary file 1 [file nutrients-12-01509-s001.pdf]

nutrients

Supplemental Material

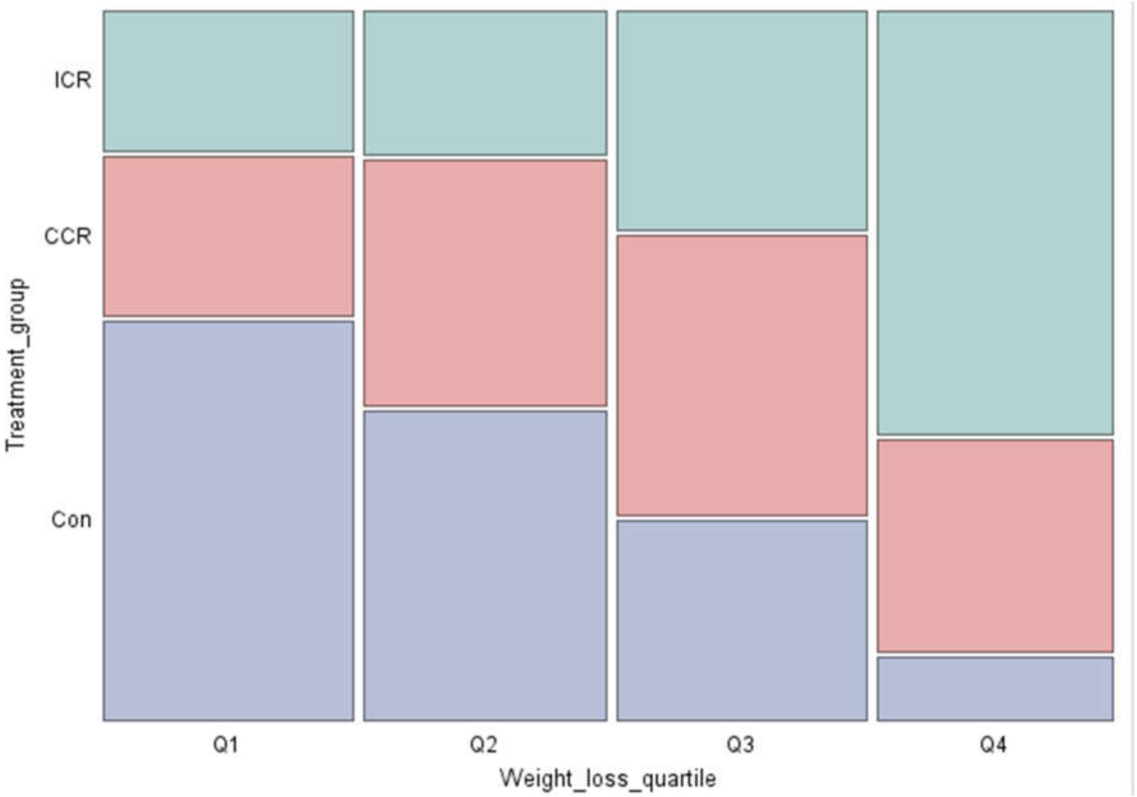

Figure 1. Distribution of the three original study arms (ICR, CCR, controls) among the four weight loss quartiles after 12 weeks. CCR, continuous calorie restriction; Con, controls; ICR, intermittent calorie restriction.

More participants of the intervention groups ICR and CCR are found in Q4 in comparison to Q1, while the control group is found more often in Q1. The number of participants of ICR and CCR are increasing from Q1 over Q2 and Q3 to Q4, while the numbers of participants of the control groups is decreasing from Q1 to Q4. For detailed information regarding the original study arms and the study objectives and results of the original study see our previous publication by Schuebel et al. [1].

Table 1. Mean values and relative change (%) of BMFC week 12 and week 50 across the study arms ICR, CCR, and controls compared with the baseline values, n = 137.

		**Baseline Mean ± SD**	**Week 12 Mean ± SD**	**Log_e relative change (Baseline – Week 12) Mean ± SEM**	***p*-value**	**Week 50 Mean ± SD**	**Log_e relative change (Baseline – Week 50) Mean ± SEM**	***p*-value**
**BMFC [%]**	ICR	42.4 ± 9.8	40.4 ± 11.1	-5.9 ± 2.9	<0.01*	43.0 ± 9.6	1.3 ± 2.0	0.4
	CCR	42.6 ± 7.3	42.0 ± 8.2	-2.3 ± 1.9		42.0 ± 8.2	-3.0 ± 1.9	
	Con	44.1 ± 8.9	42.4 ± 8.7	-4.3 ± 2.0		43.5 ± 8.4	-1.8 ± 2.0	

* p= significant at the 0.05 level (two tailed). Data are shown as mean ± SD and as mean ± SEM for week 12 and week 50 for Log_e relative change with baseline values as reference. *p*-values of the four weight loss quartiles were calculated with linear mixed models adjusted for sex and age (baseline to week 12, baseline to week 50). Abbreviations: BMFC, bone marrow fat content; CCR, continuous calorie restriction; Con, controls; ICR, intermittent calorie restriction; SD, standard deviation; SEM, standard error of the mean.

Even though there was a tendency for greater weight loss with ICR vs. CCR vs. control (see supplemental table S 1/figure S 1), there were no differential effects on BMFC across trial arms.

Thus, statistical adjustment of analyses on weight loss and BMFC for initial trial arms did not affect the present associations.

References

1. Schubel R, Nattenmuller J, Sookthai D, Nonnenmacher T, Graf ME, Riedl L, Schlett CL, von Stackelberg O, Johnson T, Nabers D *et al*: **Effects of intermittent and continuous calorie restriction on body weight and metabolism over 50 wk: a randomized controlled trial**. *The American journal of clinical nutrition* 2018, **108**(5):933-945.
